# ZNF281 is recruited on DNA breaks to facilitate DNA repair by non-homologous end joining

**DOI:** 10.1038/s41388-019-1028-7

**Published:** 2019-09-30

**Authors:** Sara Nicolai, Robert Mahen, Giuseppe Raschellà, Alberto Marini, Marco Pieraccioli, Michal Malewicz, Ashok R. Venkitaraman, Gerry Melino

**Affiliations:** 1 Medical Research Council, Toxicology Unit, University of Cambridge, Leicester, LE1 9HN UK; 2 Medical Research Council, Cancer Unit, University of Cambridge, Cambridge, CB2 0XZ UK; 3 Laboratory of Biosafety and Risk Assessment, ENEA, 00123 Rome, Italy; 4 Department of Experimental Medicine, University of Rome Tor Vergata, 00133 Rome, Italy

**Keywords:** Cancer therapy, Non-homologous-end joining, Non-homologous-end joining, Cancer therapy, Non-homologous-end joining

## Abstract

Efficient repair of DNA double-strand breaks (DSBs) is of critical importance for cell survival. Although non-homologous end joining (NHEJ) is the most used DSBs repair pathway in the cells, how NHEJ factors are sequentially recruited to damaged chromatin remains unclear. Here, we identify a novel role for the zinc-finger protein ZNF281 in participating in the ordered recruitment of the NHEJ repair factor XRCC4 at damage sites. ZNF281 is recruited to DNA lesions within seconds after DNA damage through a mechanism dependent on its DNA binding domain and, at least in part, on poly-ADP ribose polymerase (PARP) activity. ZNF281 binds XRCC4 through its zinc-finger domain and facilitates its recruitment to damaged sites. Consequently, depletion of ZNF281 impairs the efficiency of the NHEJ repair pathway and decreases cell viability upon DNA damage. Survival analyses from datasets of commonly occurring human cancers show that higher levels of ZNF281 correlate with poor prognosis of patients treated with DNA-damaging therapies. Thus, our results define a late ZNF281-dependent regulatory step of NHEJ complex assembly at DNA lesions and suggest additional possibilities for cancer patients’ stratification and for the development of personalised therapeutic strategies.

## Introduction

The maintenance of genome stability is of paramount importance for cell viability, as cells are constantly exposed to endogenous and exogenous hazards that undermine DNA integrity and function. Cells utilise a complex and coordinated mechanism collectively known as the DNA damage response (DDR) to reverse any damage that hinders replication or alters the encoded information [1, 2]. Many anti-tumour therapies, such as chemo- or radiotherapy, exploit DNA damage to induce cell cycle arrest and eventually cell death in the rapidly dividing cancer cells [3]. The cellular response to DNA damage is therefore a key factor in determining patients’ outcome following treatments and is strictly dependent on the efficiency of the repair machinery [4–7]. Accordingly, the altered expression and functions of DDR factors can lead to resistance or hypersensitivity to cancer therapies. Hence, a deep understanding of the molecular mechanisms that govern DNA repair is relevant to improve the effectiveness of anti-tumour therapies and to develop novel targeted strategies.

DNA double-strand breaks (DSBs), such as breaks caused by ionising radiation (IR), are the most lethal type of DNA damage cells must address. Homologous recombination (HR) and non-homologous end joining (NHEJ) are the two main cellular mechanisms used to repair DSBs [8]. While the former is a slow and accurate process restricted to the late S and G2 phases of the cell cycle, the latter is a faster but error-prone mechanism that represents the most frequently used pathway in the cell. In addition to the main DDR factors, accessory components include several transcription factors that fine-tune the core machinery through poly-ADP ribose polymerase (PARP)-dependent chromatin remodelling [9].

While the main molecular components of the DDR have been identified, recent evidence has revealed the participation of new factors. For example, PAXX (paralog of XRCC4 and XLF) has been discovered as a new component of the DDR [10], reviving the search for additional regulators [11]. The transcription factor ZNF281 (zinc finger protein 281) has been described to play a fundamental role in controlling cellular stemness [12–15]. Consistent with this, murine embryos lacking the homolog Zfp281 (zinc finger protein 281) die between E7.5 and E8.5, implying a key developmental function for this gene [12]. In addition, ZNF281 induces epithelial-mesenchymal transition (EMT) in tumour cells and controls the expression of several key EMT-associated genes [16]. Furthermore, in our previous studies, we found that ZNF281 antagonises the differentiation of murine cortical neurons and neuroblastoma cells [17] and that it is indirectly involved in the DDR, promoting the expression of several DDR genes in response to DNA-damaging drugs [18]. However, the role of ZNF281 within the DDR remains largely uncharacterised and poorly understood.

Here, we report findings that implicate ZNF281 as a direct participant in NHEJ. Following genotoxic stress, ZNF281 is rapidly recruited to DNA lesions and physically interacts with core components of the NHEJ repair machinery. ZNF281 facilitates the recruitment of XRCC4 (X-Ray Repair Cross Complementing 4) to DSBs, thus supporting the proper execution of the DNA repair process. Consistently, ZNF281-deficient cells challenged with IR show decreased viability and persistent activation of the DDR signalling cascade. In line with this, survival analysis of cancer patients treated with DNA damaging therapies reveals that higher expression of ZNF281 correlates with poor prognosis, suggesting that ZNF281-mediated DNA repair may favour chemoresistance. Our findings provide important new insight into the molecular events that mediate DNA repair by NHEJ on damaged chromatin.

## Results

### ZNF281 is rapidly recruited to DNA damage sites

**Fig. 1:**
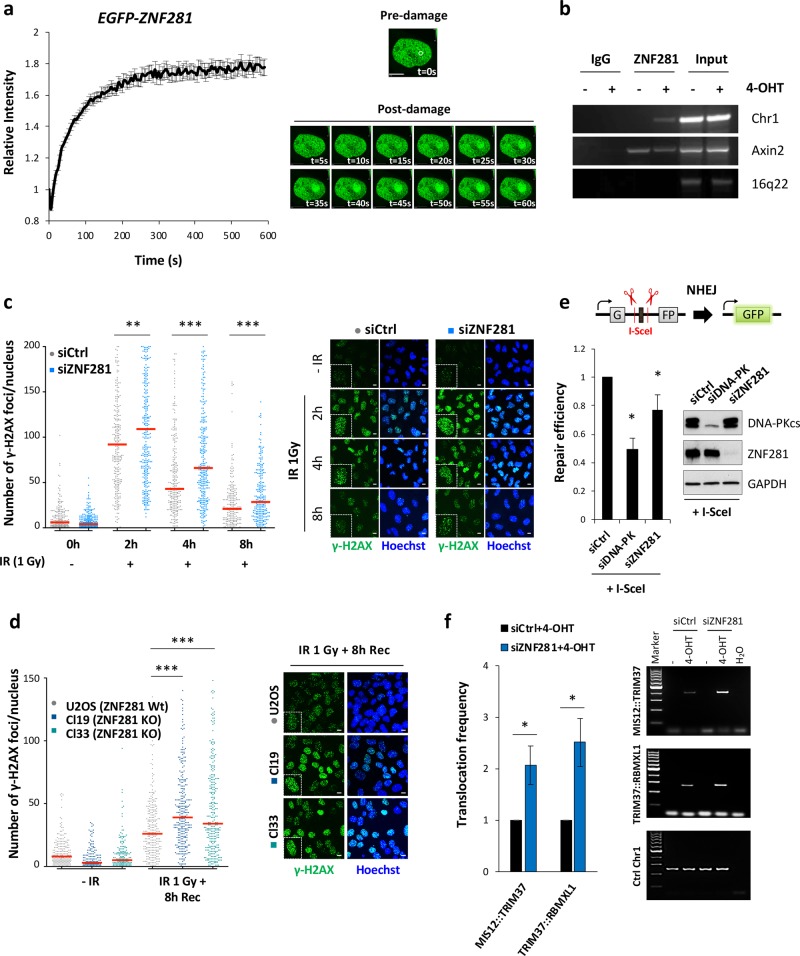
ZNF281 depletion impairs the DNA damage response and suppresses NHEJ. **a** EGFP–ZNF281 recruitment kinetics at DNA damage sites induced by a 355 nm laser in U2OS cells. Data were obtained from 75 cells from three independent experiments. Graphs present means ± SEM. Representative time-lapse images of the first 60 s are shown on the right. The white circle indicates the irradiated area. Scale bars, 10 μm. **b** ChIP analysis in U2OS-I-PpoI cells indicating the recruitment of ZNF281 to site-specific DNA break on chromosome 1 generated by I-PpoI upon activation with 1 μM 4-OHT for 2 h. Axin2 and 16q22 were used as positive and negative controls for ZNF281 ChIP, respectively; *n* = 2. **c** Number of γ-H2AX foci in U2OS cells, in which ZNF281 expression was depleted after exposure to 1 Gy of IR. After treatment, cells were allowed to recover for the indicated times and then fixed for immunofluorescence staining. Images of 200–300 cells were acquired at each time point from three biological replicates. The number of γ-H2AX foci per nucleus was automatically counted and plotted as individual data points (left panel); red lines represent medians; **p < 0.01, ***p < 0.001 (Mann–Whitney non-parametric test). Representative images are shown in the right panel. Scale bars, 10 μm. **d** Immunofluorescence staining for γ-H2AX in U2OS cells and in two ZNF281 knockout clones. Cells were exposed to 1 Gy of IR and then fixed after 8 h of recovery. Data obtained from at least 200 cells are plotted as individual points representing the number of γ-H2AX foci per nucleus (left panel). Red lines represent medians; *n* = 3; ***p < 0.001 (Mann–Whitney non-parametric test). Representative immunofluorescence images are shown (right panel). Scale bars, 10 μm. **e** Schematic of NHEJ reporter system (top). The presence of an adenoviral exon disrupts the GFP ORF, thus inactivating the gene. Cleavage by I-SceI and subsequent repair through the NHEJ pathway reconstitute functional GFP that is measured using FACS. Scrambled siRNA-transfected U2OS harbouring integrated NHEJ reporter cassette were used as control to measure the relative repair efficiency of DNA-PKcs- and ZNF281- depleted cells (left). Graphs present means ± SD; *n* = 3; *p < 0.05 (two-tailed Student’s *t*-test). Western blot showing the knockdown efficiency of DNA-PKcs and ZNF281 (right); GAPDH was used as a loading control. **f** Translocation assay in control or ZNF281-depleted DIvA cells. Upon AsiSI induction with 4-OHT, MIS12::TRIM37 and TRIM37::RBMXL1 rejoining frequencies were detected by qPCR (left panel) or end-point PCR (right panel). Amplification of an AsiSI-free region was used as a DNA input control. Graphs present means ± SEM; *n* = 5; *p < 0.05 (two-tailed Student’s *t*-test)

A common feature of DDR factors is their rapid mobilisation on chromatin following DNA damage. Thus, we verified the presence of the ZNF281 protein at DNA lesions to establish a direct role of ZNF281 within the DDR. We used laser microirradiation coupled with live cell imaging to create localised DSBs in single cells expressing EGFP–ZNF281 and followed the relocalisation process in real time. ZNF281 was recruited to the UV-induced DNA lesions and to the surrounding area within the first few seconds after damage, and its signal remained stable at these sites for several minutes (Fig. 1a). We further revealed the physical relocalisation of ZNF281 to DSBs using the I-PpoI system [19]. Here, the ectopic expression of the inducible endonuclease I-PpoI results in site-specific DNA cleavage on a single site on chromosome 1 and on multiple sites on the ribosomal DNA (Supplementary Fig. S1a). The resulting DSBs recapitulate all the key aspects of the normal DDR, as evidenced by ATM auto-phosphorylation and 53BP1 foci formation (Supplementary Fig. S1b). Chromatin immunoprecipitation of ZNF281 after 4-hydroxytamoxifen (4-OHT)-mediated I-PpoI activation revealed that ZNF281 only bound to the I-PpoI cut site on chromosome 1 after DSB induction (Fig. 1b). As expected, ZNF281 binding to the promoter of one of its known target genes (i.e. Axin2 [16]) was not affected by the 4-OHT treatment (Fig. 1b). Taken together, these results provide strong evidence for the prompt recruitment of ZNF281 to DNA damage sites, suggesting the potential involvement of this factor in the DNA repair process.

To test this hypothesis, we analysed the kinetics of changes in the levels of the phosphorylated histone variant γ-H2AX in cells depleted for ZNF281 expression and challenged with IR. The number of γ-H2AX foci scored after treatment with 1 Gy of IR was significantly greater in ZNF281-silenced cells than in control cells at all recovery times analysed (Fig. 1c). Importantly, the increased levels of γ-H2AX, which served as a read-out of unrepaired damage, observed following the knockdown of ZNF281 with a siRNA, were recapitulated in two knockout clones generated using CRISPR/Cas9 (Clustered Regularly Interspaced Short Palindromic Repeats/CRISPR-associated protein 9) technology (Fig. 1d). Furthermore, we obtained similar results by irradiating cells with a higher dose of IR (5 Gy) and detecting γ-H2AX levels using western blot analysis (Supplementary Figs. S1c and S1d). Altogether, these data reveal the persistent and prolonged activation of the DNA damage signalling pathway in the absence of ZNF281 after a genotoxic insult.

### ZNF281 deficiency compromises DNA repair

Since X-ray exposure induces many types of DNA damage including the potentially lethal DSBs, we sought to determine the effect of ZNF281 depletion on the two major repair pathways involved in repairing DSBs, i.e. the NHEJ and HR pathways. Therefore, we used U2OS cell lines stably expressing reporter cassettes for NHEJ (Fig. 1e) and HR (Supplementary Fig. S1e). Transfection of the I-SceI endonuclease in these cells induced DNA breaks in both constructs and the subsequent repair was measured using FACS analysis. Although depletion of ZNF281 reduced the repair efficiency of both NHEJ (Fig. 1e) and HR (Supplementary Fig. S1e), a consistent and statistically significant impairment was observed only for the NHEJ repair pathway. In line with a possible involvement of ZNF281 in NHEJ, we detected a strong increase of its signal in the chromatin-bound fraction immediately after IR exposure, with a similar kinetics of NHEJ core factors, such as DNA-PKcs and the heterodimer Ku (Supplementary Fig. S1f).

Next, we evaluated the DSB repair process in the absence of ZNF281 using the DIvA (DSB inducible via AsiSI) cell line [20]. Here, similar to the I-PpoI system, the 4-OHT-mediated activation of the endonuclease AsiSI results in multiple site-specific DNA breaks scattered all over the genome. Using a recently developed assay [21] we measured the illegitimate rejoining frequencies of distant AsiSI-induced DSBs between sites on the same chromosome (MIS12::TRIM37) or on different chromosomes (TRIM37::RBMXL1) (Fig. 1f and Supplementary Fig. S1g). ZNF281 knockdown caused an increased rate of both translocation events suggesting that ZNF281 counteracts the generation of incorrect repair products. The evidence of a defective and delayed DNA repair, together with the hyperactivation of the DDR signalling of cells lacking ZNF281 expression suggest a direct role for this factor in the repair process.

### ZNF281 interacts with NHEJ factors

**Fig. 2:**
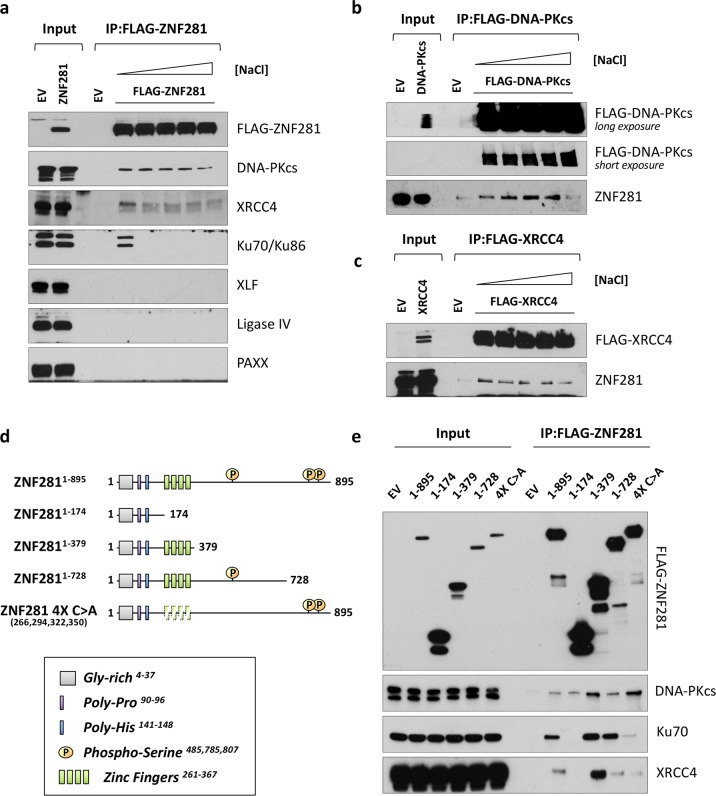
ZNF281 interacts with components of the NHEJ repair complex. **a** Co-immunoprecipitation of ZNF281 with NHEJ components in HEK293T cells transfected with the FLAG-ZNF281 expression plasmid. **b**, **c** HEK293 cells stably expressing the FLAG-DNA-PKcs construct (**b**) or transiently transfected with the FLAG-XRCC4 plasmid (**c**) were used to co-immunoprecipitate endogenous ZNF281. **a**–**c** Increasing concentrations of salt (ranging from 150 to 600 mM NaCl) were used during the wash steps to gradually increase the stringency as a read-out of the strength of the protein–protein interactions. **d** Schematic showing the full-length and mutant FLAG-tagged ZNF281 constructs. Characterized domains and relevant post-translational modifications are listed in the box on the bottom. **e** Mapping of ZNF281 domains that interacted with its protein partners. ZNF281 constructs were transfected into HEK293T cells and analysed using Co-IP

As we observed a greater effect of ZNF281 depletion on the NHEJ pathway than on the HR pathway (Fig. 1e), we focused first on the role of ZNF281 in NHEJ. We analysed whether ZNF281 directly interacts with any of the NHEJ complex core components to establish the mechanism by which ZNF281 affects the efficiency of the NHEJ pathway. Therefore, we performed co-immunoprecipitation (CoIP) experiments of FLAG-tagged proteins and detected strong, salt-resistant interactions of ZNF281 with XRCC4 and DNA-PKcs (the catalytic subunit of the DNA-PK subcomplex), and salt-sensitive interactions with the cofactors Ku70 and Ku86 (Fig. 2a–c). Importantly, these interactions were also confirmed at the endogenous level and in different cell lines (Supplementary Fig. S2a, b).

We generated a series of deletion mutants and used them as bait in CoIP experiments to identify the domain of ZNF281 interacting with XRCC4 and the DNA-PK complex (Fig. 2d, e). ZNF281 bound XRCC4 and Ku through its zinc-finger domain, while DNA-PKcs appeared to interact with many regions throughout the ZNF281 protein. In fact, while full-length ZNF281 (ZNF281^1–895^) co-immunoprecipitated with all interactors, the ZNF281^1–174^ mutant failed to pull-down either XRCC4 or Ku, but not DNA-PKcs. Importantly, the addition of the zinc-finger domain to the ZNF281^1–174^ fragment (ZNF281^1–379^) restored these interactions. We next generated a mutant harbouring four point mutations in the zinc-finger domain (hereafter named ZNF281 4X C>A) to further confirm this finding. In this mutant, a single Cys in each zinc-finger (specified in Fig. 2d) was converted to Ala to disrupt the structure while maintaining the rest of the primary sequence. Similar to the total ablation of the domain (as shown in the ZNF281^1–174^ CoIP), the ZNF281 4X C>A mutant almost completely lost the ability to bind Ku and XRCC4, while its binding affinity for DNA-PKcs was retained (Fig. 2e).

### ZNF281 is recruited to DNA lesions through the zinc-finger domain and its relocalisation is impaired upon PARP inhibition

We next sought to identify the molecular mechanism underlying ZNF281 relocalisation to DNA damage sites. We based the investigation on the evidence that ZNF281 is phosphorylated by the DDR kinases ATM and ATR upon DNA damage induction [22]. In a large-scale proteomic analysis by Matsuoka et al., phosphorylation on S/T-Q sites (consensus for ATM and ATR) after irradiation was shown to be a common feature of many DDR factors, and the authors identified three of these residues in the ZNF281 protein [22] (Fig. 2d). Thus, we hypothesised that these phosphorylation events might trigger ZNF281 recruitment to damaged chromatin. We measured the recruitment of the non-phosphorylatable mutants S785A and S807A (in which Ser residues were converted to Ala), showing that single mutations of these sites were not sufficient to prevent ZNF281 relocalisation to damaged DNA (Supplementary Figs. S3a, b). On the other hand, a modest defect in the ability of ZNF281 to be retained at DDR sites was achieved through the simultaneous inactivation of both residues (in the double mutant S785A + S807A) (Supplementary Fig. S3c). Based on these findings, the phosphorylation of these sites potentially plays a role in modulating ZNF281 function in the DDR. However, we cannot exclude the existence of additional, as yet uncharacterised phosphorylation sites that also participate in this regulatory mechanism.

**Fig. 3:**
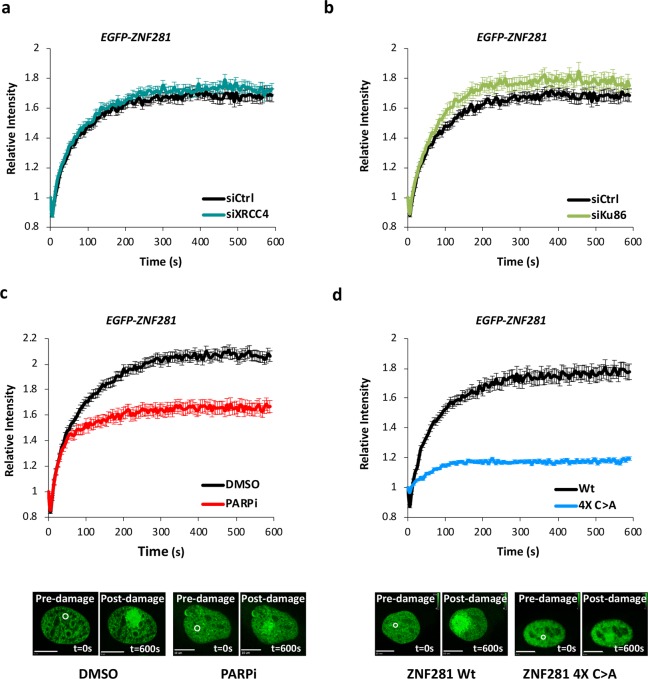
ZNF281 recruitment to DNA lesions depends on PARP. **a**, **b** U2OS cells stably expressing EGFP–ZNF281 were treated with siRNAs targeting XRCC4 (**a**), Ku86 (**b**) or DNA-PK-cs (Supplementary Fig. S3d). ZNF281 relocalisation to the UV-induced DNA lesions in silenced cells was measured and compared to siCtrl-treated cells. Fifty to 75 cells were analysed at each time point in three independent experiments. Graphs present means ± SEM. siCtrl-treated cells were plotted separately against individual siRNA-treated cells to clearly present the data. **c** U2OS–EGFP–ZNF281 cells were pre-treated with 10 μM PARPi (Olaparib) for 1 h before laser microirradiation. Data were obtained from 60 to 75 cells in three independent experiments. Graphs present means ± SEM. **d** Recruitment of ZNF281 wild-type (Wt) and ZNF281 4X Zinc mutant (4X C>A) to UV laser-induced DNA damage sites in U2OS cells. Eighty cells expressing the ZNF281 4X C>A mutant were analysed from three independent experiments. Mean values ± SEM are plotted against the ZNF281 Wt curve (from Fig. 1a) for comparison. Representative images acquired before and after damage are shown below the graphs of the quantitative analysis in panels **c** and **d**. White circles indicate the irradiated area. Scale bars, 10 μm

As the formation of DNA repair foci is an ordered and hierarchical process, in which early recruited factors promote the binding of later components, we next asked whether ZNF281 recruitment was mediated by one of the newly described interactors XRCC4, Ku, or DNA-PKcs (Fig. 2). We treated cells with siRNAs targeting these factors and we detected the ZNF281 fluorescent signal at the laser-induced damage sites to test this hypothesis (Fig. 3a, b and Supplementary Fig. S3d, e). Notably, while the DNA-PK subcomplex acts as a sensor of DSBs in the first stages of the NHEJ process, XRCC4 (together with XLF and Ligase IV) is involved in the final ligation step. Thus, ZNF281 must act upstream of XRCC4 at an earlier stage, since XRCC4 depletion had no effect on ZNF281 relocalisation (Fig. 3a). Surprisingly, the knockdown of Ku (Fig. 3b) and DNA-PKcs (Supplementary Fig. S3d) also failed to block ZNF281 recruitment, and even induced a slight increase. Next, we explored the dependency of ZNF281 recruitment on PARP activity. Indeed, poly ADP-ribosylation (parylation) mediated by PARPs enzymes (mostly PARP-1) is one of the initial events in the DDR pathway [23, 24]. Interestingly, transcription factors seem to be recruited at the site of damaged DNA in a PARP-dependent manner to promote chromatin decondensation in the surrounding area [9], increasing the accessibility of the damaged DNA to repair factors. Consistent with these findings, we observed a substantial decrease in ZNF281 intensity and spreading at the laser-induced damage sites upon PARP-1 inhibition (Fig. 3c and Supplementary Fig. S3f).

Furthermore, we observed a severe defect in the relocalisation of the ZNF281 4X C>A mutant to UV-induced damage sites (Fig. 3d). Disruption of this domain not only causes almost complete ablation of ZNF281 recruitment but also loss of its characteristic spreading outwards from the initially damaged area (Fig. 3d). Altogether, these data point to a dual role for the zinc-fingers in mediating both ZNF281 interactions and relocalisation to damage sites. However, the recruitment defect of the ZNF281 4X C>A mutant is most likely due to the intrinsic DNA binding ability [25] of this region rather than to the disruption of ZNF281 interactions, as evidenced by XRCC4 and Ku knockdown experiments (Fig. 3a, b).

### ZNF281 promotes the recruitment of XRCC4 to DNA damage sites

**Fig. 4:**
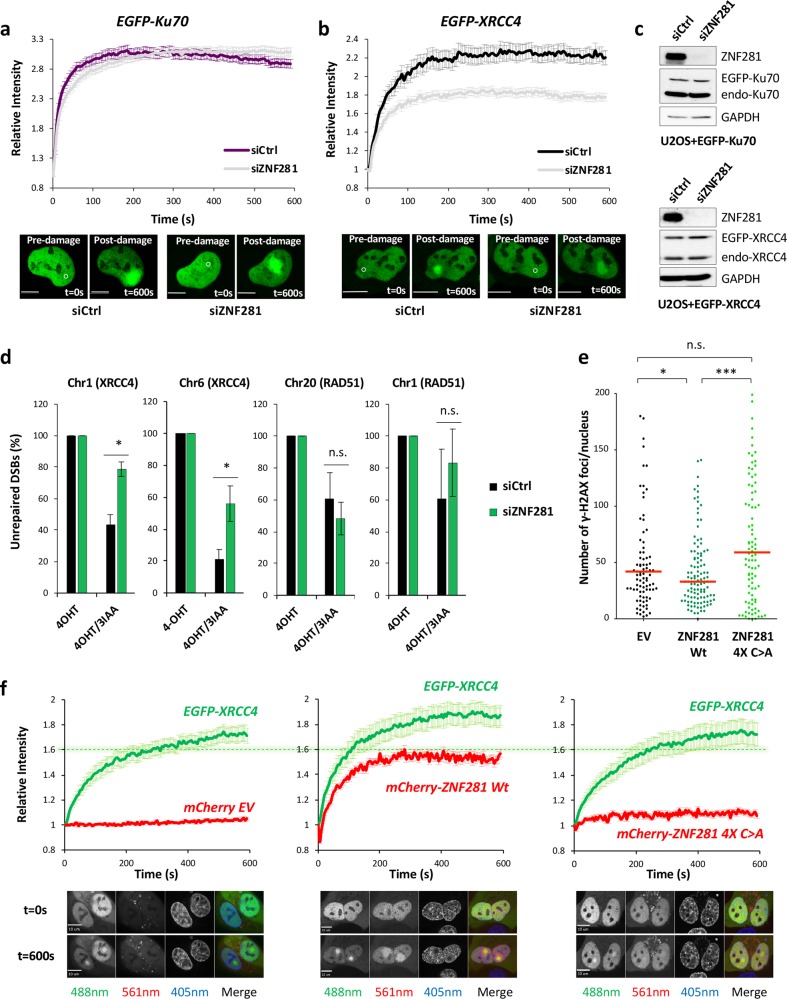
ZNF281 promotes the recruitment of XRCC4 to DNA lesions. **a**, **b** Stable U2OS–EGFP–Ku70 (**a**) or U2OS–EGFP–XRCC4 (**b**) cells were transiently transfected with an siRNA to silence ZNF281 expression and the recruitment kinetics of the indicated fluorescent proteins were analysed. At least 100 cells were analysed at each time point from three biological replicates. Graphs present means ± SEM. Representative images of control and ZNF281-silenced cells are shown below each graph. White circles indicate the irradiated areas. Scale bars, 10 μm. **c** Representative WB showing the ZNF281 knockdown efficiency in U2OS–EGFP–Ku70 (top panel) or U2OS–EGFP–XRCC4 (bottom panel); GAPDH was used as a loading control. **d** Ligation-mediated cleavage assay on DIvA cells transfected with the indicated siRNAs. Cells were treated with 4-OHT (300 nM for 6 h) or with 4-OHT, followed by 3-IAA (500 μg/ml for 30 min). The percentage of unrepaired DSBs (relative to 4-OHT-treated cells) is presented. Graphs are means ± SEM; *n* = 3; *p < 0.05 (two-tailed Student’s *t*-test), n.s. not significant. **e** Number of γ-H2AX foci in U2OS ZNF281^−/−^ clone 33 complemented with a plasmid encoding ZNF281 Wt, ZNF281 4X C>A, or an empty vector (EV). Forty-eight hours after transfection, cells were irradiated with 1 Gy of IR, allowed to recover for an additional 8 h and then fixed for immunofluorescence staining. Images of ~100 cells were acquired at each experimental time point from three biological replicates. The number of γ-H2AX foci per nucleus was automatically counted and plotted as individual data points; red lines represent medians; *p < 0.05, ***p < 0.001 (Mann–Whitney non-parametric test). **f** U2OS ZNF281^−/−^ clone 33 stably expressing EGFP–XRCC4 was transiently transfected with mCherry-ZNF281 Wt or mCherry-ZNF281 4X C>A mutant and compared with mCherry empty vector-transfected cells (EV). The graph shows the fluorescence intensity of the green channel (for EGFP–XRCC4) and red channel (for mCherry-ZNF281 or EV) measured as the mean value for 60–75 cells from four independent experiments. Error bars represent the SEM. The green dotted line (set to the fluorescence intensity observed for the control at 300 s) highlights faster and greater recruitment of EGFP–XRCC4 only when cells are complemented with ZNF281 Wt. Representative images captured before (0 s) and after damage (600 s) are shown below each graph. Scale bars, 10 μm. 488 nm: EGFP–XRCC4. 561 nm: mCherry EV (left panel), mCherry-ZNF281 Wt (central panel), or mCherry-ZNF281 4X C>A (right panel). 405 nm: Hoechst

We sought to obtain additional insights into the functional role of ZNF281 in the DDR after its recruitment to DNA lesions. We postulated a mechanism by which ZNF281 facilitates the relocalisation of its interacting partners to the DNA damage sites. We initially compared the recruitment kinetics of ZNF281 with the early and later components of the NHEJ pathway, such as Ku and XRCC4, respectively, to assess this hypothesis (Supplementary Fig. S4a, b). The results highlighted a very rapid relocalisation of Ku70 (i.e. <5 s), while XRCC4 and ZNF281 displayed similar and slower kinetics (~10 s) (Supplementary Fig. S4c). Hence, we tested the effect of ZNF281 depletion on the recruitment of these two factors (Fig. 4a–c). ZNF281 knockdown partially impaired XRCC4 recruitment (Fig. 4b) and, importantly, defects in XRCC4 mobilisation were also observed in the U2OS knockout clone (Supplementary Fig. S4d). Conversely, the ZNF281 deficiency had no effect on the relocalisation of the early component Ku (Fig. 4a). Altogether, these data indicate a role for ZNF281 in promoting XRCC4 recruitment to the damaged chromatin. To confirm this finding, we used the DIvA system to measure the induction and the repair kinetics specifically at the AsiSI sites previously found enriched for XRCC4 [20]. Using the ligation-mediated cleavage assay we demonstrated that the percentage of AsiSI-induced DSBs is not affected by ZNF281 depletion (Supplementary Fig. S4e). Next, exploiting the auxin inducible degron (AID) that is fused to the AsiSI endonuclease, we could trigger AsiSI degradation with 3-Indoleacetic acid (3-IAA) and measure the repair rate at those sites. We found that ZNF281 knockdown cells showed a slower repair at 30 min after 3-IAA treatment (Fig. 4d), thus confirming the defect in DSBs repair at XRCC4-enriched sites. Interestingly, we observed that the repair kinetics of RAD51-enriched DSBs (HR prone sites) was not affected by ZNF281 depletion (Fig. 4d).

We reintroduced ZNF281 into the knockout clone and monitored the subsequent behaviour of XRCC4 (Fig. 4f). Importantly, the addition of ZNF281 Wt (wild-type) increased XRCC4 fluorescence at damage sites, indicating a rescue of its initial recruitment ability (Fig. 4f). Consistent with the restoration of XRCC4 recruitment, transfection of ZNF281 Wt also promoted faster resolution of the DNA damage, as evidenced by the decreased number of γ-H2AX foci per nucleus detected after IR treatment (Fig. 4e and Supplementary Fig. S4f). On the other hand, complementation of the knockout clone with the defective ZNF281 4X C>A mutant that fails to relocalise on DNA lesions and to bind XRCC4 was unable to restore XRCC4 recruitment (Fig. 4f) or to reverse the DNA repair defect (Fig. 4e). Notably, simultaneous imaging of XRCC4 and ZNF281 revealed only partial colocalisation of the two proteins, as XRCC4 was strictly confined to the damage sites, while ZNF281 also spread outwards from this area (Fig. 4f). The latter observation suggests that ZNF281 is potentially involved in different levels of the DDR, playing additional roles other than the sole recruitment of XRCC4.

### ZNF281 deficiency sensitises cells to IR and its expression predicts resistance to DNA damage-based cancer therapies

**Fig. 5:**
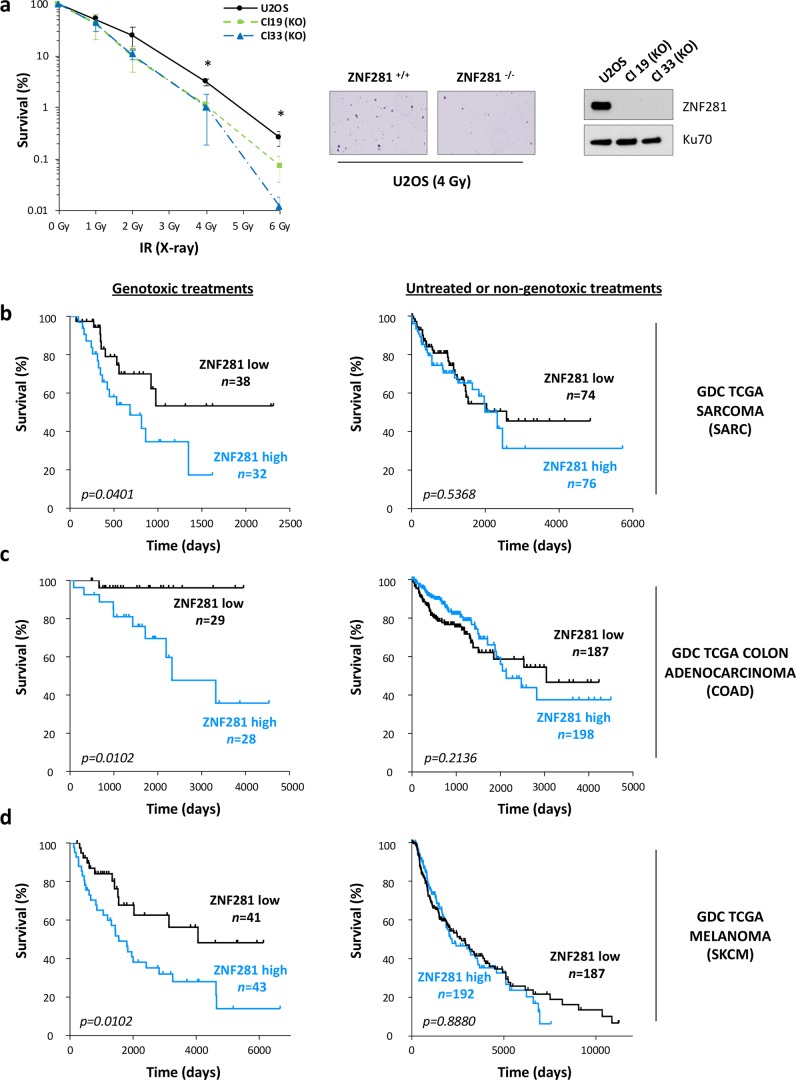
ZNF281 expression predicts sensitivity to DNA damaging therapies. **a** Clonogenic survival curves of U2OS parental cells compared with two ZNF281^−/−^ clones (Cl19 and Cl33) after IR exposure with the indicated doses of X-ray. Graph presents means ± SD; *n* = 3; *p < 0.05 (two-tailed Student’s *t*-test). Representative images are shown in the central panel. WB analysis of ZNF281 levels is shown on the right; Ku70 was used as a loading control. **b**-**d** Kaplan–Meier plots (KM) indicating the overall survival probability of patients from the indicated TCGA datasets (**b** sarcoma; **c** colon adenocarcinoma; **d** melanoma). Left KM plots: patients treated with genotoxic therapies; right KM plots: untreated patients or treated with non-genotoxic therapies. The difference between the curves for ZNF281 high expressing samples (blue) and ZNF281 low expressing ones (black) are compared by Log-rank Mantel–Cox test and *p*-values are shown in each plot

Given the involvement of ZNF281 in the correct execution of the NHEJ process, we used clonogenic survival assays to assess the function of ZNF281 in protecting the cells from a genotoxic insult. Indeed, ZNF281 ablation in two independent U2OS knockout clones significantly reduced their clonogenic potential after exposure to different IR doses (Fig. 5a). These results were confirmed in HEK293T cells transiently transfected with a siRNA targeting ZNF281 (Supplementary Fig. S5a). Based on this and our previous experimental evidence [18], we explored the possibility of using ZNF281 expression levels as a predictive marker of resistance to the DNA-damaging agents commonly used as anti-tumour therapies (radio- and chemo-therapies). Hence, we analysed datasets of common human malignancies from The Cancer Genome Atlas (TCGA). Kaplan–Meier survival curves of sarcoma (Fig. 5b, left), colon adenocarcinoma (Fig. 5c, left), melanoma (Fig. 5d, left) and lung adenocarcinoma (Supplementary Fig. S5b, left) patients demonstrated that high expression of ZNF281 significantly correlates with poor prognosis only in patients treated with genotoxic agents. Conversely, ZNF281 expression is not predictive for survival probability of untreated patients or of those treated with non-genotoxic therapies (Fig. 5b–d and Supplementary Fig. S5b, right). Altogether, these data suggest that ZNF281 should be evaluated as a novel marker to predict response to cancer treatments based on DNA damage induction, in line with a direct role of ZNF281 in the DDR also in pathological contexts.

## Discussion

Results presented here underscore a non-canonical role of the zinc finger protein ZNF281 in the execution of the DDR. Indeed, we show that ZNF281 is rapidly recruited to sites of DNA damage through its zinc finger domain, with a mechanism that is partially dependent on PARP activity. ZNF281 supports DNA repair mainly via NHEJ facilitating the recruitment of the core NHEJ factor XRCC4 to the sites of DNA damage. Accordingly, loss of ZNF281 sensitises cells to IR and affects DNA repair efficiency. In addition, translocation assay data indicate that illegitimate rejoining events occur more frequently in absence of ZNF281, suggesting that ZNF281 depletion could contribute to genomic instability. These observations demonstrate that the activity of ZNF281 in DDR goes well beyond its previously described role of transcriptional activator of XRCC4 after DNA damage [18].

NHEJ is initiated by recognition of DSBs by Ku dimer. Binding of Ku to exposed DNA ends at DSBs triggers allosteric conformational change that allows Ku to recruit downstream NHEJ repair components [1]. Although Ku binding occurs very rapidly after DNA damage induction (Supplementary Fig. S4a), the recruitment of XRCC4 is slower (Supplementary Fig. S4b) presumably as it is acting at the final ligation stage of the NHEJ repair reactions. In agreement with its role in recruiting XRCC4, ZNF281 and XRCC4 display remarkably similar kinetics of binding to the DNA damage sites (Supplementary Fig. S4c). Thus, our data identify an additional level of control of NHEJ machinery assembly through ZNF281-assisted XRCC4 loading on DSBs.

Laser microirradiation experiments reveal that ZNF281 relocalisation to DNA damage sites depends on (i) the presence of its DNA binding domain (zinc finger domain) and (ii) partially on PARP activity (Fig. 3c, d). The addition of PAR chains (parylation) in the proximity of the damaged regions assembles a docking station that recruits other DDR factors in an orderly fashion to accurately execute the repair process [26]. Interestingly, many transcription factors that bind to DNA breaks require PARP activity for proper localization [9] and recently, a proteome-wide approach has revealed that ZNF281 itself is parylated in response to different genotoxic insults [27], suggesting that this post-translational modification plays a role in the regulation of ZNF281 activity. However, it is not clear yet whether the impairment of ZNF281 recruitment following PARP inhibition is only due to the decreased parylation on ZNF281 residues or to the disruption of ZNF281 interactions with other parylated proteins, such as histones or DDR factors. In this regard, no PAR-binding domains have been identified on ZNF281 protein so far and further studies are needed to better clarify the mechanisms through which parylation is involved during ZNF281 relocalisation. Overall, our findings suggest that PARP inhibition might prevent ZNF281 recruitment through multiple but not mutually exclusive mechanisms, i.e. by affecting ZNF281 parylation and/or chromatin/DDR factors parylation.

Given the involvement of poly-ADP-ribose, it is likely that chromatin remodelling participates in the late XRCC4 stabilisation at DSBs. It has previously been observed that XRCC4 binding to DSBs requires partial nucleosome reorganisation [28]. Indeed, a dynamic series of events takes place to modulate chromatin structure in order to properly execute DNA double-stand break repair [29, 30]. One of these mechanisms involve the chromatin factor HP1-β that promotes chromatin alterations to facilitate H2AX phosphorylation [31]. An intriguing hypothesis is that ZNF281 is also involved in the chromatin changes that normally follow DNA injury. Previous report [13, 14] demonstrated that Zfp281 recruits the NuRD repressor complex to the Nanog promoter to block its expression during somatic cell reprogramming. Similarly, in the context of DNA damage, ZNF281 might participate in the recruitment of this complex to specifically prevent transcription at DNA damage sites. Notably, HDAC1 and HDAC2 (histone deacetylases of the NuRD complex) promote DNA repair through the NHEJ pathway, while their depletion causes hypersensitivity to DNA-damaging agents [32], similar to the hypersensitivity elicited by a ZNF281 deficiency. Since Zfp281 binds HDAC2 on Nanog [13] and TET2 promoters [14], we can speculate that ZNF281 acts together with members of the NuRD complex for the chromatin remodelling necessary for the proper execution of NHEJ. Thus, it is conceivable that ZNF281 somehow contributes to NHEJ possibly by promoting transient repressive chromatin configurations occurring during DSB repair [33]. The latter would be an additional function of ZNF281 on DNA damage sites beside loading XRCC4.

ATM/ATR-mediated phosphorylation directly affects DDR, as also recently shown by the ATM-dependent recruitment at DNA damage sites of ZNF506, a member of the zinc finger protein family like ZNF281 [34]. Although it has been previously shown that ZNF281 requires phosphorylation by the kinases ATM and ATR upon DNA damage [22], our data demonstrate that mutation of two candidate target residues S785 and S807 did not affect the ability of ZNF281 to localise to the sites of DNA damage. Further studies are necessary to fully understand the role of ZNF281 phosphorylation in DDR, as previously unidentified phosphorylated residues might play a crucial function in its regulation.

DNA repair is important in physiological as well as in pathological contexts. Over the years, a growing effort has been made to increase the success of conventional and new anticancer treatments. In this regard, it has been recently reported that inhibition of the DNA repair machinery’s core component DNA-PK enhances the efficiency of anticancer strategies relying on oncolytic viruses [35]. Likewise, an intriguing prediction of our study is that inhibition or inactivation of ZNF281 could slow down DNA repair during anticancer therapy with DNA damaging agents, thus increasing their effectiveness. Conversely, a high expression of ZNF281 could favour the survival of tumour cells subjected to genotoxic therapies, thus triggering a process of chemoresistance and tumour regrowth. Indeed, bioinformatic analysis of cancer patients treated with genotoxic therapies demonstrates that low expression of ZNF281 is associated with better prognosis, while patients with elevated levels of ZNF281 have a decreased survival probability (Fig. 5). These results suggest that ZNF281 levels could be used to predict patients’ responsiveness to DNA-damaging treatments. In line with this finding, another zinc finger protein, ZNF830, has been recently implied in the chemoresistance to genotoxic therapies, as a consequence of its role in promoting DNA repair by HR [36]. Furthermore, the strong correlation between ZNF281 recruitment and PARP activity (Fig. 3c) could be exploited to improve genotoxic therapies efficiency. It is, indeed, tempting to speculate that ineffective DNA-damaging treatments could be implemented with PARP inhibitors in order to sensitise ZNF281 highly expressing tumours that show resistance to genotoxic therapies alone.

DNA repair following a genotoxic insult occurs through complex mechanisms, in which essential core factors work in close connection with a yet undefined number of other factors that act in the optimization of the process [11]. In this context, the activity of ZNF281 should be included as an additional step for docking the XRCC4 core factor to the damaged sites and possibly in creating a suitable chromatin setting for carrying out DNA repair. While further work is required to understand the mechanisms that control the access of ZNF281 to the sites of damage and to uncover other interactors, our data identify a novel player in DNA repair whose dysfunction may have detrimental effects on genomic stability.

## Materials and methods

### Cell lines and CRISPR/Cas9 KO generation

U2OS and HEK293T cell lines (ATCC) were cultured in DMEM supplemented with 10% fetal bovine serum, 100 U/ml penicillin, 0.1 mg/ml streptomycin at 37 °C in 5% CO_2_ and 100% humidity.

U2OS ZNF281 knockout clones were generated with the Edit-R CRISPR-Cas9 Gene Knockout system (Dharmacon). Briefly, U2OS cells were transfected with ZNF281 crRNA (#CR-006958-04), tracrRNA (#U-002005) and hCMVCas9 plasmid (#U-005100, Puro^R^) for 24 h and then selected with 4 μg/ml of puromycin. After 24 h of selection, puromycin was washed away and surviving cells were allowed to recover for additional 48 h. Cells were then seeded at low density to isolate single clones. The presence of insertions/deletions causing frameshift in both alleles was confirmed by sequencing.

The 293 H clone stably expressing physiological levels of FLAG-DNA-PKcs was described in [37].

U2OS (or U2OS CRISPR clones) stably expressing EGFP–Ku70, EGFP–XRCC4 or EGFP–ZNF281 (Wt and mutants), were obtained through antibiotic selection with 800 μg/ml of G418 for 2 weeks. Clones were then pooled on a single population to avoid clonal heterogeneity.

U2OS harbouring integrated HR and NHEJ reporter cassettes were a kind gift of Martin Bushell (Beatson Institute, Glasgow, UK).

U2OS stably expressing the ER–AID–AsiSI endonuclease (DIvA cells) [20] were a generous gift of Gaëlle Legube (Center for Integrative Biology, Toulouse, FR).

### Cloning and mutagenesis

pEGFP-C1-FLAG-Ku70 (Addgene plasmid, #46957) and pEGFP-C1-FLAG-XRCC4 (Addgene plasmid, #46959) were a gift from Steve Jackson [38]. pEGFP-C1-ZNF281 and mCherry-C1-ZNF281 were sub-cloned from pcDNA-ZNF281-FLAG.

Site-directed mutants of pcDNA-ZNF281-FLAG, pEGFP-C1-ZNF281 or mCherry-C1-ZNF281 were generated using the QuikChange II Site-Directed Mutagenesis Kit (Agilent #200523) (for phosphomutants) or with the QuikChange Multi Site-Directed Mutagenesis Kit (Agilent #200514) (for the zinc-fingers mutant).

Expression vectors with different domains of ZNF281 were obtained by molecular cloning from pcDNA-ZNF281-FLAG plasmid.

The sequence of all plasmids generated was checked by sequencing. All the primers used for cloning and mutagenesis are listed in the Supplementary Table S1.

### Treatments and transfections

Transfections were carried out using Lipofectamine 2000 or Lipofectamine LTX (Invitrogen) following manufacturer’s instruction. For knockdown experiments, cells were transfected by using Lipofectamine RNAiMAX (Invitrogen) according to the manufacturer’s instructions. siRNAs targeting the following proteins were purchased from Dharmacon: ZNF281 (SMARTpool, L-006958); DNA-PKcs (SMARTpool, L-005030); BRCA1 (SMARTpool, J-003461); XRCC4 (SMARTpool, L-004494). Knockdown of Ku86 was performed by using a pool of two custom siRNAs purchased from Sigma (target sequences: GAAGUUCUGUCACAGCUGAUU; AAGCGAGUAACCAGCUCAUAAUU). A non-targeting siRNA (4390844, Ambion) was used as negative control in all knockdown experiments.

X-ray irradiation was performed using the Xstrahl RS320 machine.

Induction of DSBs in I-PpoI and AsiSI systems was carried out by treatment with 4-hydroxytamoxifen (4-OHT) at the indicated times and doses (Sigma, H7904). Degradation of the AsiSI endonuclease was induced by treatment with 3-Indoleacetic acid (3-IAA, auxin) (ChemCruz).

The specific PARP inhibitor Olaparib was used at 10 μM for 1 h before laser microirradiation or western blot analysis.

Complete information about ‘Materials and methods' is reported in the Electronic Supplementary Material. All the antibodies used are listed in the Supplementary Table S2. Uncropped scans of all western blots are shown in Supplementary Figs. S6 and S7.

## Supplementary information

Supplementary Information
