# Supplementary material for: ZNF281 is recruited on DNA breaks to facilitate DNA repair by non-homologous end joining
Source: Oncogene. 2019 Sep 30;39(4):754–66. doi: 10.1038/s41388-019-1028-7 (PMC6976523; doi:10.1038/s41388-019-1028-7)
Supplement: Supplementary file 1 — Supplementary Information [file 41388_2019_1028_MOESM1_ESM.pdf]

Supplementary Information for

ZNF281 is recruited on DNA breaks to facilitate DNA repair by non-homologous end joining

Sara Nicolai[1], Robert Mahen[2], Giuseppe Raschellà[3], Alberto Marini[1], Marco Pieraccioli[4], Michal Malewicz[1], Ashok R. Venkitaraman[2], and Gerry Melino[1,4,*]

[1] Medical Research Council, Toxicology Unit, University of Cambridge, Leicester, LE1 9HN, UK

[2] Medical Research Council, Cancer Unit, University of Cambridge, Cambridge, CB2 0XZ, UK

[3] Laboratory of Biosafety and Risk Assessment, ENEA, Rome, 00123, Italy

[4] Department of Experimental Medicine, University of Rome Tor Vergata, Rome, 00133, Italy

* To whom correspondence should be addressed. Tel: +39 06 72596976; Fax: +39 06 72596977; Email: gm614@mrc-tox.cam.ac.uk

This PDF file includes: Supplementary Figures S1-S7

Supplementary Tables S1-S2

Supplementary Materials and Methods

Supplementary References

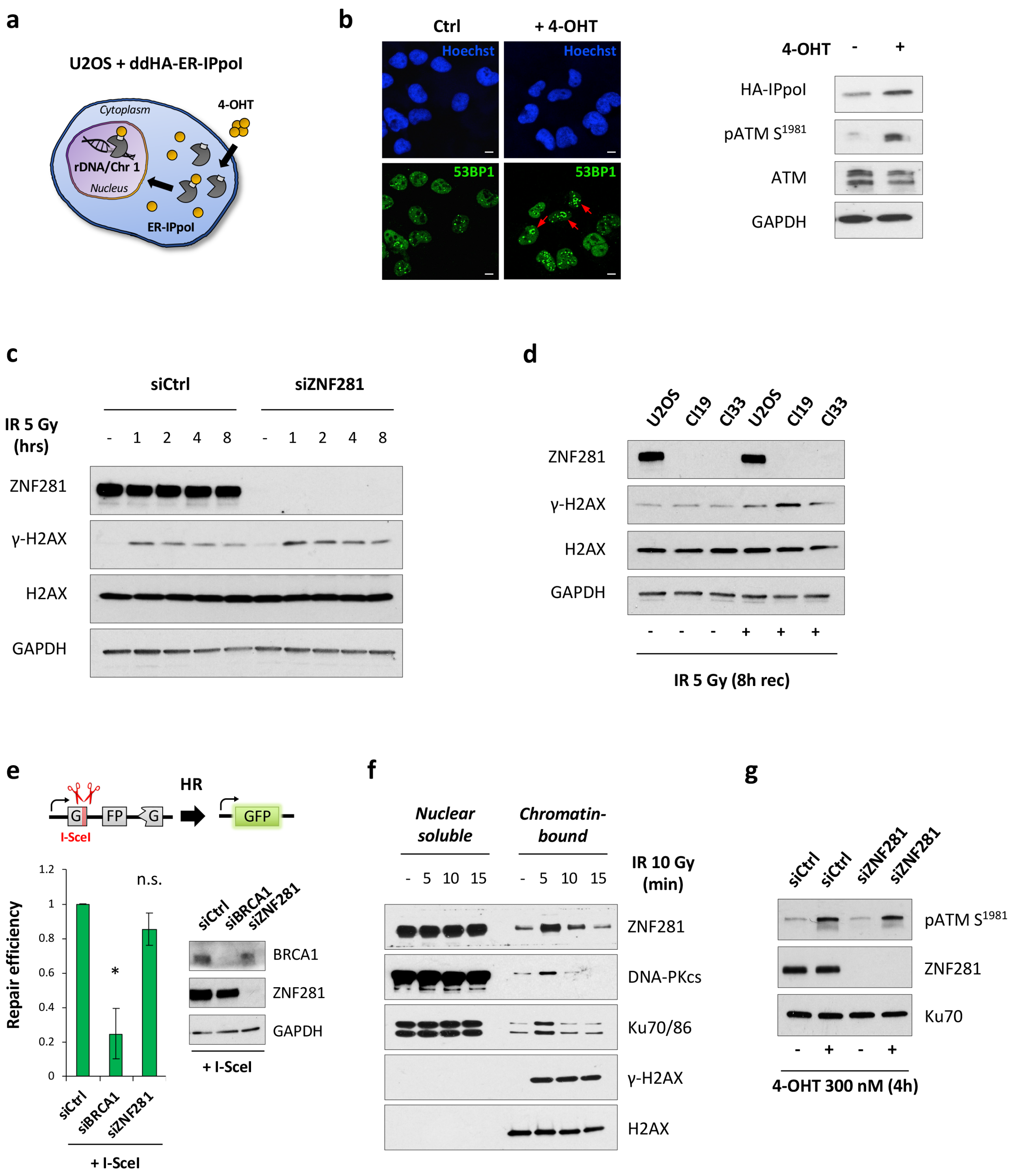

Supplementary Figure S1. (**a**) Cartoon depicting the I-PpoI system. U2OS cells were infected with a lentivirus expressing ddHA-ER-I-PpoI endonuclease. The addition of 4-hydroxytamoxifen (4-OHT) promotes the translocation of the restriction enzyme to the nucleus and induction of site-specific DSBs (on Chromosome 1 and on the rDNA). (**b**) Immunofluorescence staining for 53BP1 (*left panel*) and western

blot analysis (*right panel*) showing the correct activation of the DNA damage signalling cascade in response to 4-OHT-mediated I-PpoI activation. Red arrows showing 53BP1 foci around nucleoli indicate the induction of DNA damage in the rDNA. Scale bars, 10 μm. (**c**) Representative WB of U2OS cells exposed to 5 Gy of IR and harvested at the indicated time points. GAPDH was used as a loading control. (**d**) Representative WB of U2OS and two ZNF281$^{-/-}$ clones exposed to 5 Gy of IR and harvested 8 hours later. GAPDH was used as loading control. (**e**) Schematic depicting the HR reporter system (*top*). The HR cassette carries two mutated copies of the GFP gene, both of which are inactive. Upon I-SceI digestion, the functional GFP gene is restored by gene conversion through HR and is quantified using FACS analysis. Scrambled siRNA-transfected U2OS harbouring integrated HR reporter cassette were used as control to measure the relative repair efficiency of BRCA1- and ZNF281- depleted cells (*left*). Graphs present means ± SD; *n*=3; * $p<0.05$ (two-tailed Student's t-test), n.s. not significant. Western blot showing the knock-down efficiency of BRCA1 and ZNF281 (*right*); GAPDH was used as a loading control. (**f**) WB of sub-cellular fractions of U2OS cells exposed to 10 Gy of IR and then harvested at the indicated time points of recovery. γ-H2AX was used as control for DNA damage induction, while the amount of total H2AX shows the separation of the chromatin-bound fraction from the soluble fraction. (**g**) WB of DIvA cells (relative to Fig. 1f) demonstrates ZNF281 knockdown efficiency and pATM S^{1981} activation in response to 4-OHT treatment; Ku70 was used as a loading control.

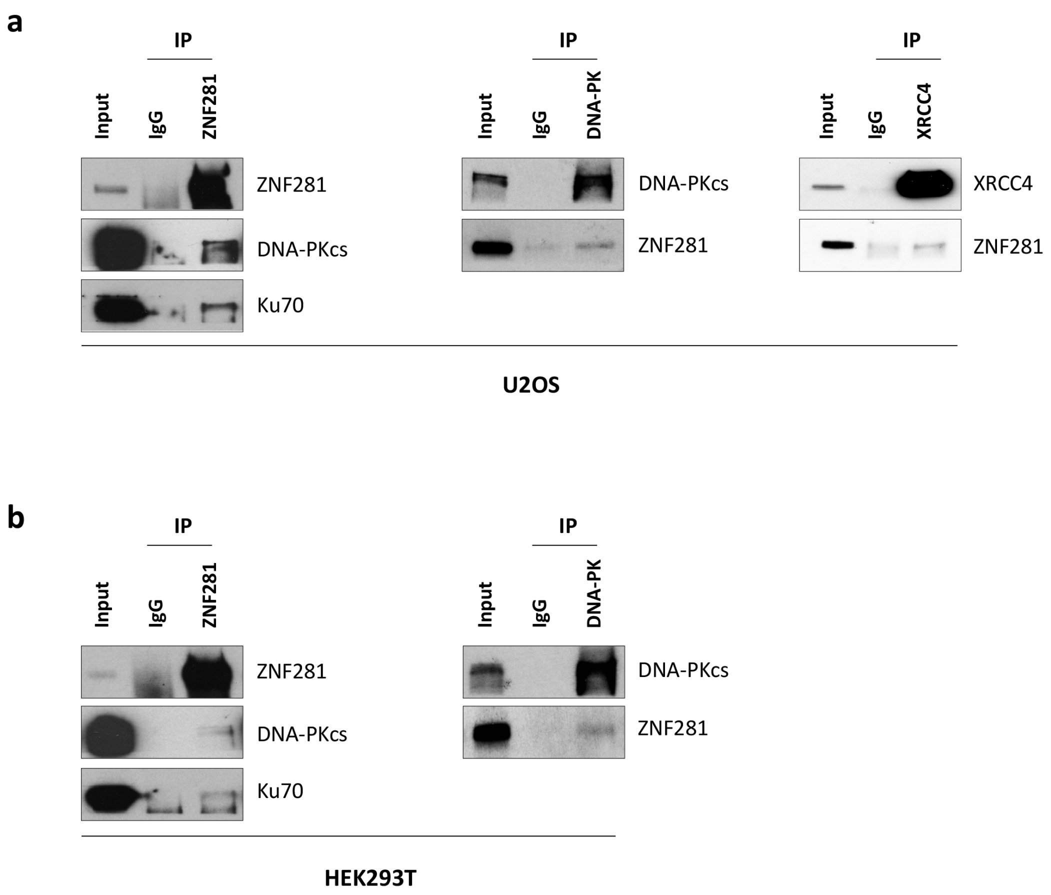

Supplementary Figure S2. (**a** and **b**) Co-IPs of the indicated endogenous proteins in U2OS (**a**) and HEK293T (**b**) cell lines.

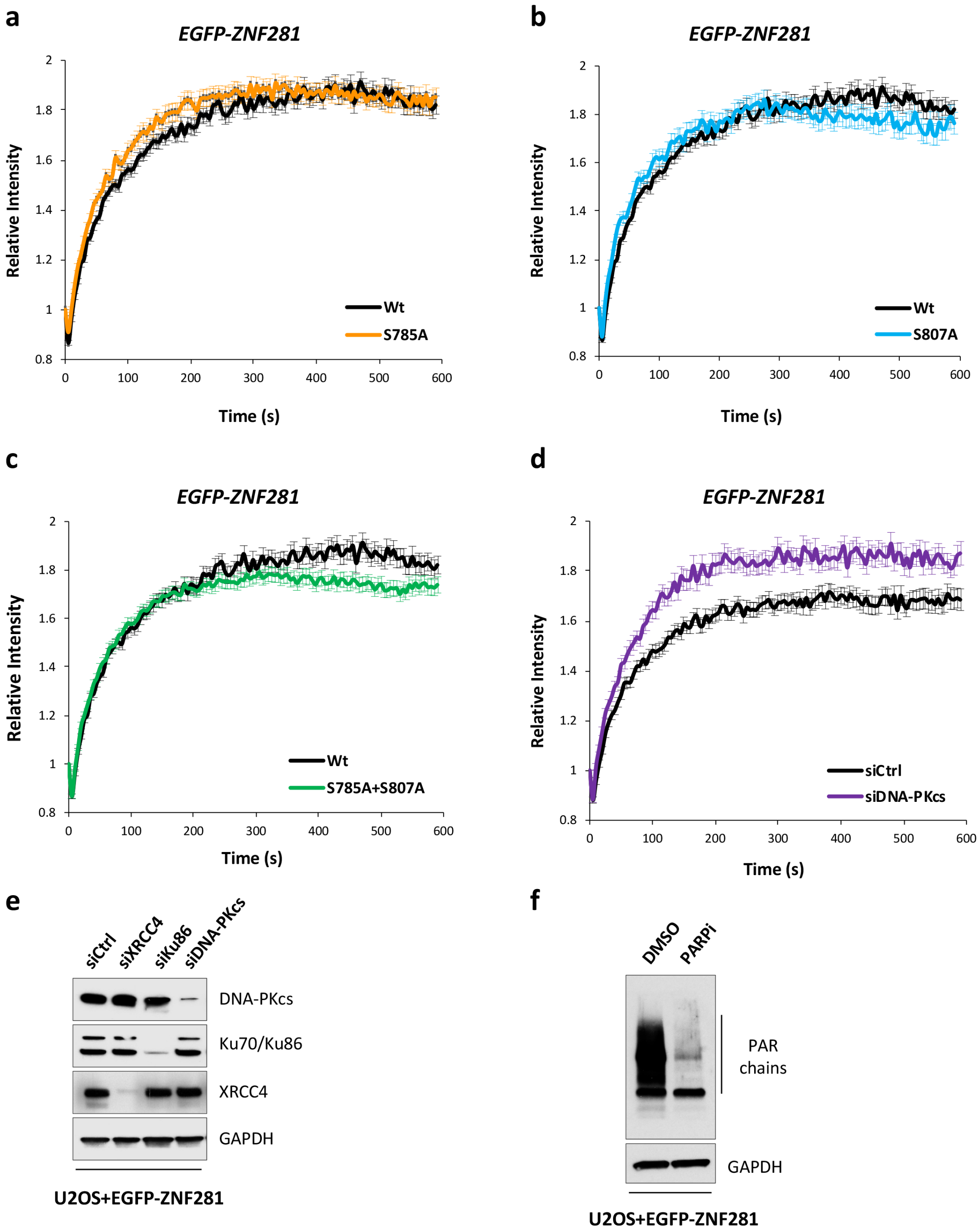

Supplementary Figure S3. (**a**, **b** and **c**) The recruitment of ZNF281 Wt to UV laser-induced DNA damage sites was compared to S785A (**a**), S807A (**b**) and S785A+S807A (**c**) phospho-mutants in U2OS cells. The ZNF281 Wt curve was repeated in each graph and plotted with individual ZNF281 mutants to clearly present the data. Data collected from 75-100 cells are presented as mean values in the graphs. Error bars represent the SEM; *n*=3. (**d**) U2OS-EGFP-ZNF281 were transfected with the indicated siRNAs. The graph shows ZNF281 recruitment measured as mean values ± SEM; *n*=3. (**e**) WB evaluating the knock-down

efficiency of the indicated proteins; GAPDH was used as loading control. This analysis is relative to experiments shown in Fig. 3a, 3b and Supplementary Fig. S3d. (**f**) WB (relative to Fig. 3c) using an antibody recognizing PAR-chains revealing the PARP inhibitor efficiency; GAPDH was used as a loading control.

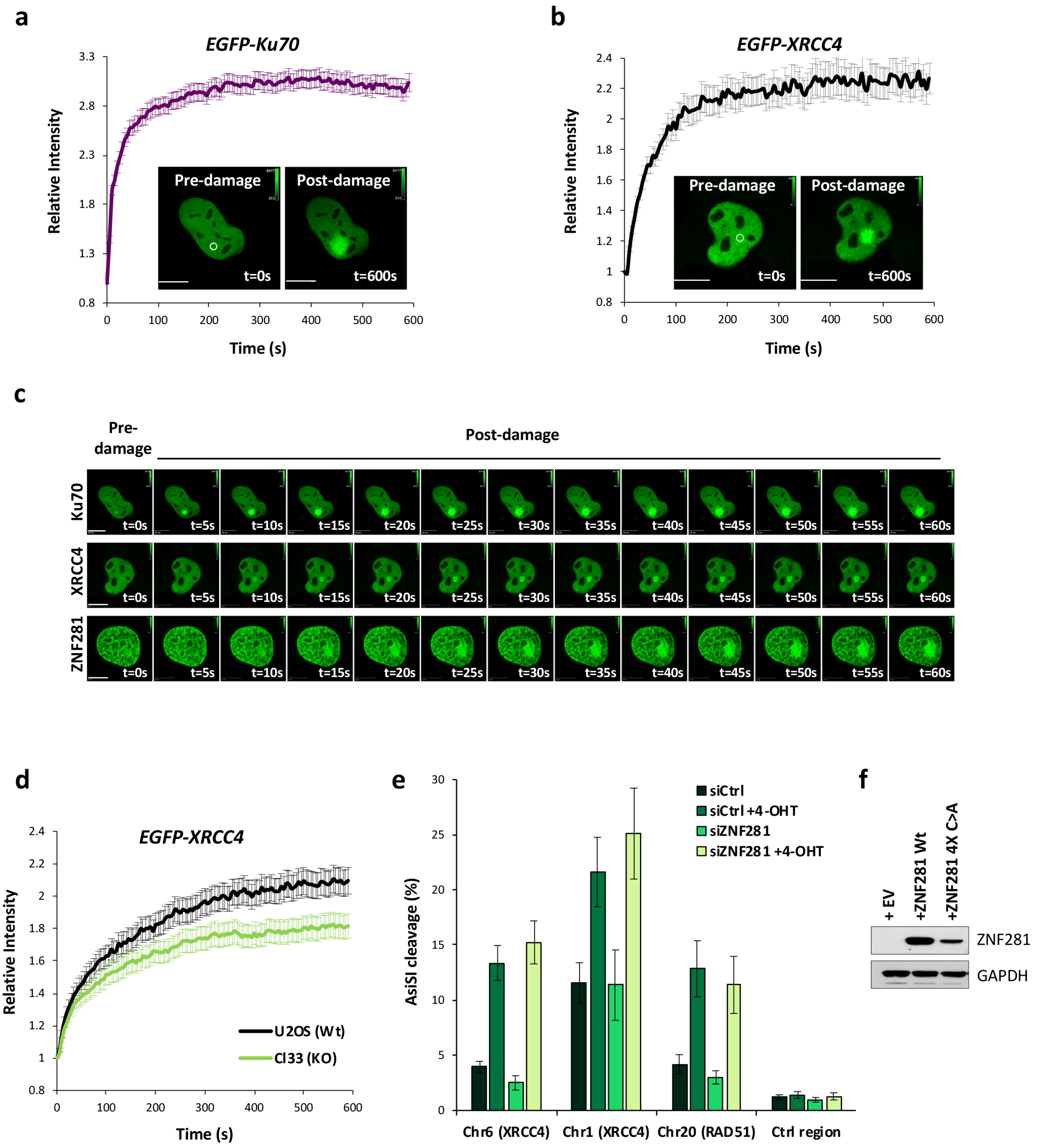

Supplementary Figure S4. (**a** and **b**) Quantification of EGFP-Ku70 (**a**) and EGFP-XRCC4 (**b**) recruitment to damage sites in U2OS stably expressing these constructs. Seventy to 90 cells were analysed from at least two independent experiments. Graphs present means ± SEM. Representative images captured before and after damage are shown. White circles indicate the irradiated areas. Scale bars, 10 μm. (**c**) Representative time-lapse images of the first 60 seconds of EGFP-tagged Ku70, XRCC4 and ZNF281 recruitment in U2OS cells following the induction of DNA damage. Scale bars, 10 μm. (**d**) Relocalisation

upon laser-induced DNA damage of EGFP-XRCC4 in parental U2OS cells and in the ZNF281 knock-out clone 33. Approximately 100 cells were analysed for each condition in three independent experiments. Graphs present means ± SEM. (**e**) Ligation-mediated cleavage assay on DIvA cells transfected with the indicated siRNAs. The percentage of AsiSI-induced cuts upon treatment with 4-OHT 300 nM for 4h is shown for each DSB. XRCC4 or RAD51 in brackets refers to a previous classification of these sites, where it was evaluated the enrichment ratio of RAD51 vs XRCC4 at each AsiSI site [1]. The specificity of the assay is demonstrated by the lack of signal in a region far from any AsiSI site (Ctrl region). (**f**) WB on U2OS ZNF281$^{-/-}$ clone 33 stably expressing EGFP-XRCC4 (relative to Fig. 4e) verifies the transient overexpression of the indicated proteins; GAPDH was used as loading control.

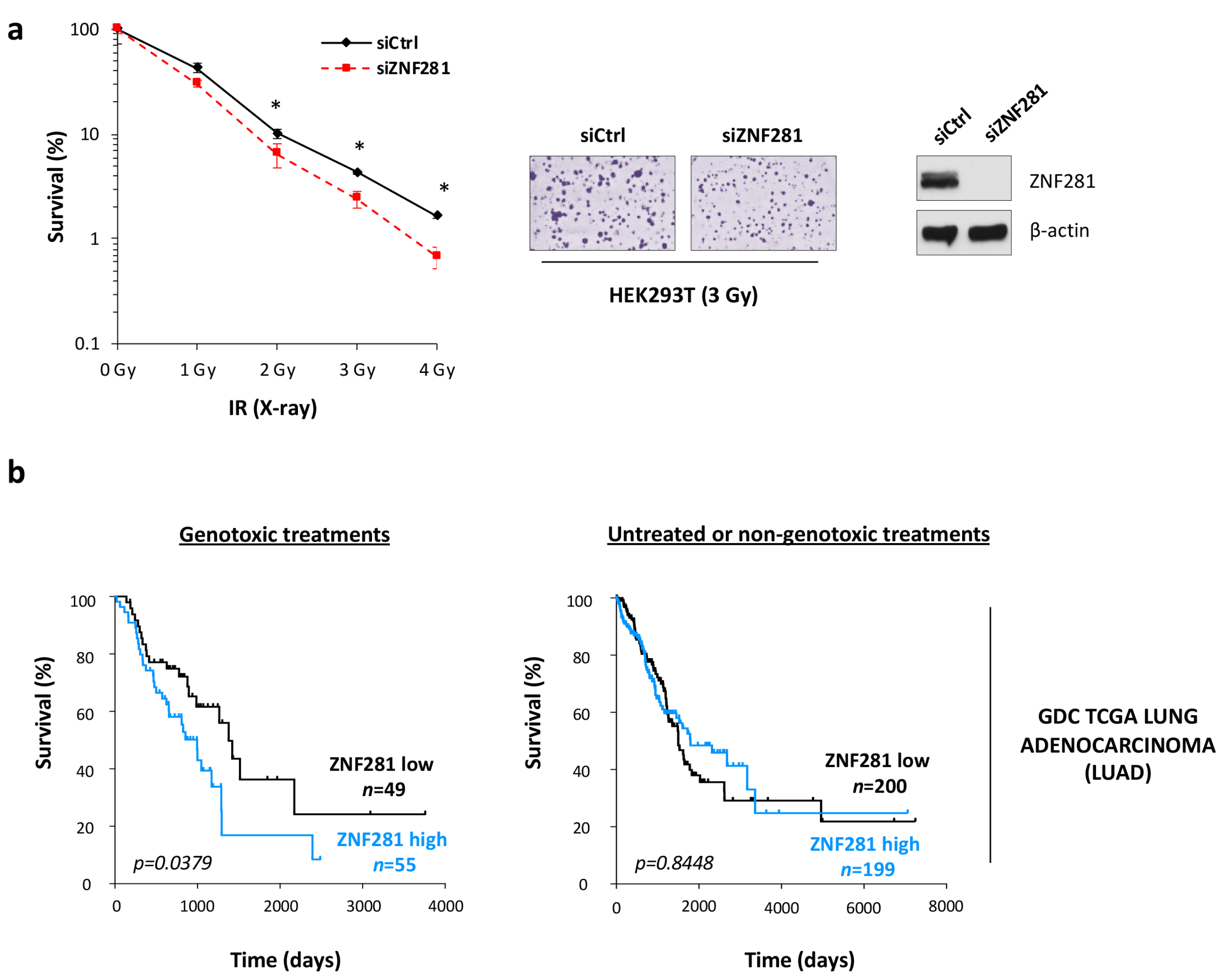

Supplementary Figure S5. (**a**) Clonogenic survival curves of HEK293T cells silenced for ZNF281 expression upon IR treatment with the indicated doses. Graph presents means ± SD; *n*=3; * p<0.05 (two-tailed Student's *t*-test). Representative images are shown in the *central panel.* WB analysis of ZNF281 levels is shown on the *right*; β-actin was used as a loading control. (**b**) Kaplan-Meier plots (KM) indicating the overall survival probability of patients from the TCGA lung adenocarcinoma dataset. *Left KM plot*: patients treated with genotoxic therapies; *right KM plot*: untreated patients or treated with non-genotoxic therapies. The difference between the curves for ZNF281 high expressing samples (blue) and ZNF281 low expressing ones (black) are compared by Log-rank Mantel-Cox test and p-values are shown in each plot.

Fig. S1c

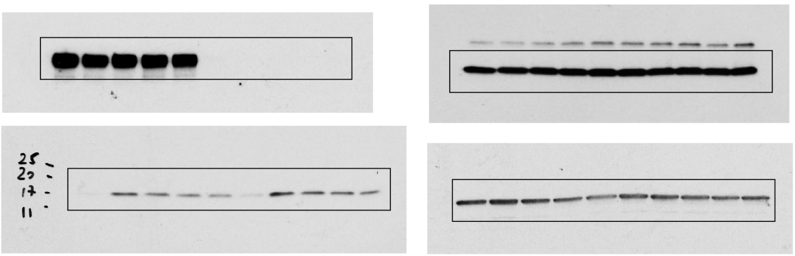

Fig. 2a

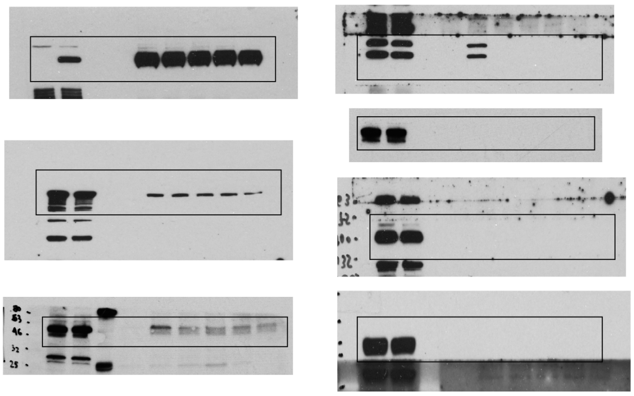

Fig. 2b

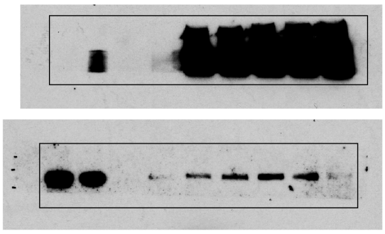

Fig. 2c

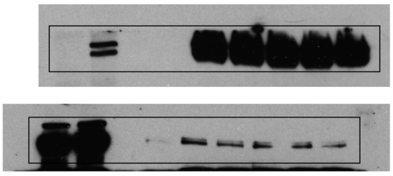

Fig. 2e

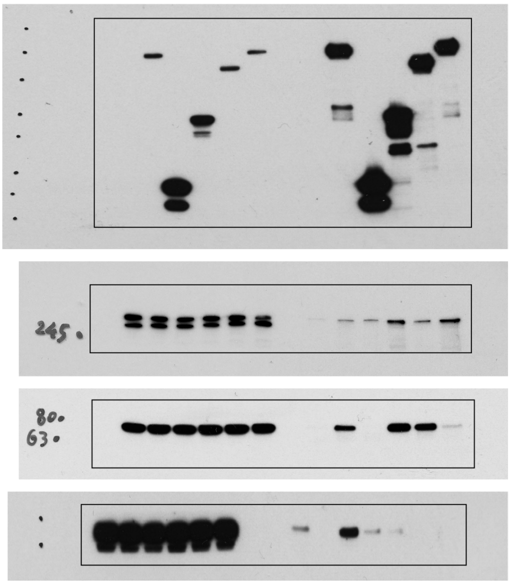

Fig. 4c

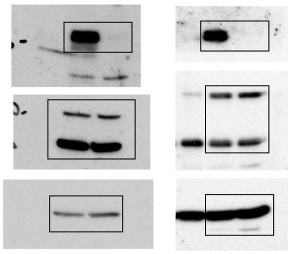

Fig. 5a

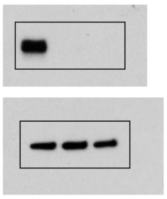

Supplementary Figure S6. Uncropped images from which WB analyses in Figures S1, 2, 4 and 5 were derived.

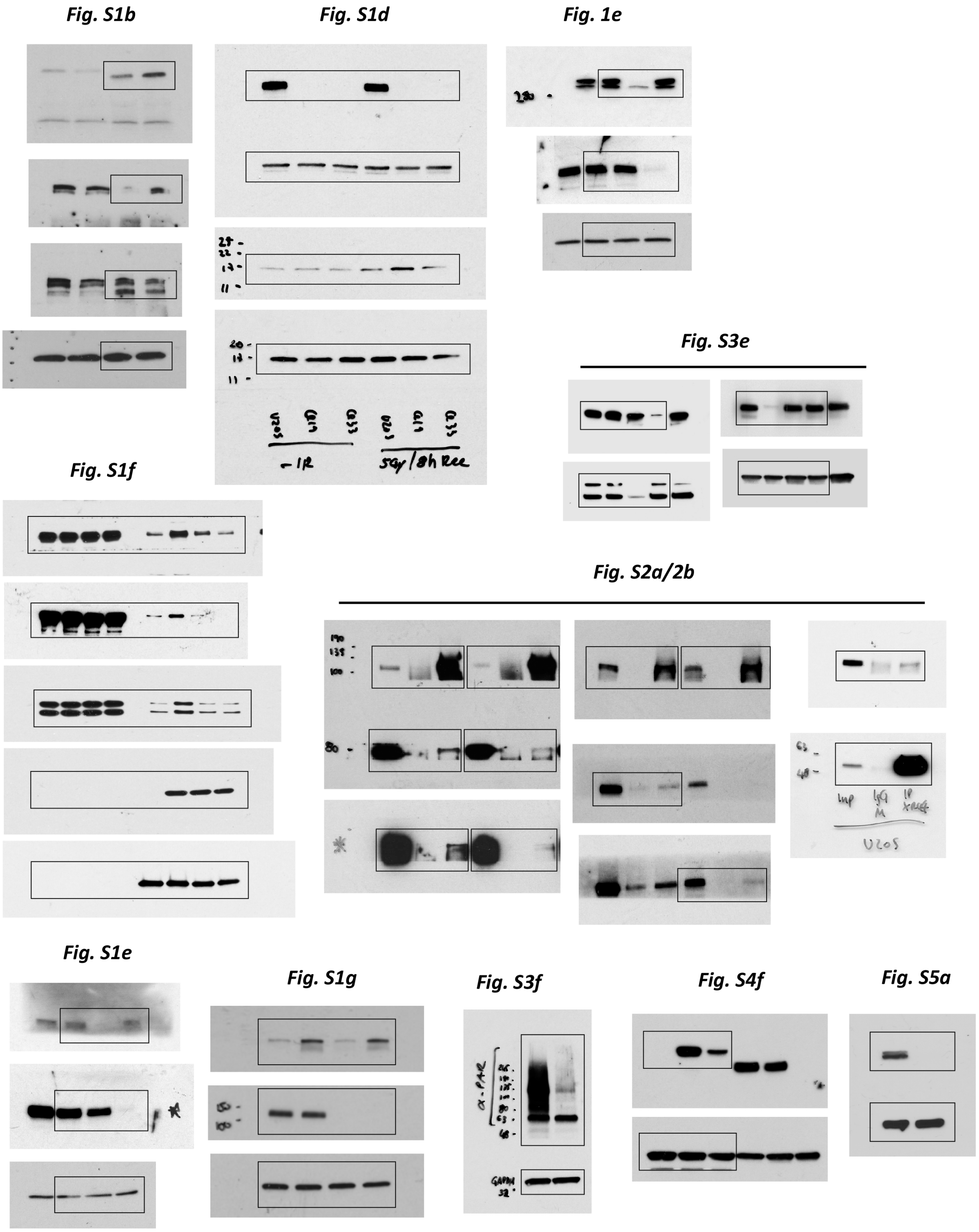

Supplementary Figure S7. Uncropped images from which WB analyses in Supplementary Figures 1, S1, S2, S3, S4 and S5 were derived.

Supplementary Table S1. List of primers

Primers used for cloning and mutagenesis	
**Name**	**Sequence**
ZNF281 XhoI Fwd	ACGACTCGAGCTATGAAAATCGGCAGTGGGTTCCTGAGTG
ZNF281 HindIII Rev	CAGGAAGCTTCGTTACCTGTAACTCTGGCTGGTGG
ZNF281$^{1\text{-}895,\ 1\text{-}174,\ 1\text{-}379,\ 1\text{-}728}$ Fwd	ATCGCCACCATGAAAATCGGCAGTGGGTTCCTGAGTGGCGGCGGA
ZNF281$^{1\text{-}174}$ Rev	ATCCTACTTGTCATCGTCGTCCTTGTAGTCGATGACGCCGTGACTCCCCC
ZNF281$^{1\text{-}379}$ Rev	ATCCTACTTGTCATCGTCGTCCTTGTAGTCTTCTGCACTAGTGGCTCCTT
ZNF281$^{1\text{-}728}$ Rev	ATCCTACTTGTCATCGTCGTCCTTGTAGTCCAACACTGAGGTAACAGATTG
S785A Fwd	CAGCCTTCCAGTCCTCAGCCCAGAAATTGACTAGCCAG
S785A Rev	CTGGCTAGTCAATTTCTGGGCTGAGGACTGGAAGGCTG
S807A Fwd	CAACAGGCTTTCAGATTCCAGCCCAGGAGTTAGCTAGCCAG
S807A Rev	CTGGCTAGCTAACTCCTGGGCTGGAATCTGAAAGCCTGTTG
C266A	CATATCTGTGATCACGCTAGTGCTGCTTTCCG
C294A	CCAGTGCAGCCAGGCTAGTATGGGTTTCATTC
C322A	GGATGTGATCAGGCCAGCATGAAGTTTATTCAG
C350A	GCCATATAAGTGTGACACTGCCCAACAGTATTTTTC
**Primers used for ChIP**	
**Name**	**Sequence**
I-PpoI cut site Chr1 Fwd	TGCTGCTTTTTCTTCTTCTCC
I-PpoI cut site Chr1 Rev	CTTCTTTCCCACCAAGTCTTC
Axin2 ZNF281BS Fwd	CCAACTCACTCAGGGGAGAC
Axin2 ZNF281BS Rev	GATTCTTGGCACAGGCAGTAG
16q22 Negative Ctrl Fwd	CTACTCACTTATCCATCCAGGCTAC
16q22 Negative Ctrl Rev	ATTTCACACACTCAGACATCACAG
**Primers used for Translocation assay**	
**Name**	**Sequence**
MIS12_Chr17_5390209 Fwd	GACTGGCATAAGCGTCTTCG
TRIM37_Chr17_ 57184285 Rev	TCTGAAGTCTGCGCTTTCCA
TRIM37_Chr17_ 57184285 Fwd	AATTCGCAAACACCAACCGT
RBMXL1_Chr1_89433139 Rev	GCCAATGGAGTTCCCTGAGTC
Ctrl_Chr1_82844750 Fwd	AGCACATGGGATTTTGCAGG
Ctrl_Chr1_82844992 Rev	TTCCCTCCTTTGTGTCACCA
Ctrl_Chr17_9784962 Fwd	ACAGTGGGAGACAGAAGAGC
Ctrl_Chr17_9785135 Rev	CTCCATCATCGCACCCTTTG
**Primers used for Ligation assay**	
**Name**	**Sequence**
dsDNA Oligonucleotide linker Fwd	Phospho-CGCAAGCTTTAATACGACTCACTATAGGG
dsDNA Oligonucleotide linker Rev	Biot-CCCTATAGTGAGTCGTATTAAAGCTTGCGAT
HIF1α (Ctrl+) Fwd	AGCTTGCTCATCAGTTGCCA
HIF1α (Ctrl+) Rev	CCAGAAGTTTCCTCACACGC
Chr1 (XRCC4) Fwd	GATTGGCTATGGGTGTGGAC
Chr1 (XRCC4) Rev	CATCCTTGCAAACCAGTCCT
Chr6 (XRCC4) Fwd	TGCCGGTCTCCTAGAAGTTG
Chr6 (XRCC4) Rev	GCGCTTGATTTCCCTGAGT
Chr22 (XRCC4) Fwd	CCTTCTTTCCCAGTGGTTCA
Chr22 (XRCC4) Rev	GTGGTCTGACCCAGAGTGGT
Chr20 (RAD51) Fwd	CCTAGCTGAGGTCGGTGCTA
Chr20 (RAD51) Rev	GAAGAGTGAGGAGGGGGAGT
Chr1 (RAD51) Fwd	CTCTAGGCTGGAAGAGACTG
Chr1 (RAD51) Rev	AGCCCGGAACTGCCCAAATC
Chr22 (Ctrl region) Fwd	CCCATCTCAACCTCCACACT
Chr22 (Ctrl region) Rev	CTTGTCCAGATTCGCTGTGA

Supplementary Table S2. List of antibodies

Antibody	Catalog	Supplier	Application
ZNF281	*ab101318*	*Abcam*	*WB/ChIP/IP*
DNA-PKcs	*ab1832*	*Abcam*	*WB/IP*
Ku70	*sc-17789*	*Santa Cruz*	*WB*
Ku86	*sc-5280*	*Santa Cruz*	*WB*
γ-H2AX S^{139}	*05-636-I*	*Millipore*	*WB/IF*
H2AX	*ab11175*	*Abcam*	*WB*
BRCA1	*sc-6954*	*Santa Cruz*	*WB*
pATM S^{1981}	*ab81292*	*Abcam*	*WB*
ATM	*sc-23921*	*Santa Cruz*	*WB*
53BP1	*MAB3804*	*Chemicon*	*IF*
XRCC4	*a generous gift from Dik Van Gent (Erasmus University, Netherlands)*		*WB*
XRCC4	*sc-365055*	*Santa Cruz*	*IP*
XLF	*sc-166488*	*Santa Cruz*	*WB*
Ligase IV	*ab80514*	*Abcam*	*WB*
PAXX	*sc-514359*	*Santa Cruz*	*WB*
FLAG	*F3165*	*Sigma*	*WB/IF*
HA	*MMS-101P*	*Covance*	*WB*
GAPDH	*G8795*	*Sigma*	*WB*
β-actin	*A5441*	*Sigma*	*WB*
PAR	*4336*	*Trevigen*	*WB*

SUPPLEMENTARY MATERIALS AND METHODS

Clonogenic assay. HEK293T cells treated with the indicated siRNAs for 48h or U2OS parental and KO clones were irradiated with different doses of IR. Immediately after irradiation cells were trypsinized, counted and re-plated in triplicate at low density. Colonies were allowed to form for 12-16 days and then fixed in 3:1 methanol:acetic acid before staining with 0.1% (w/v) crystal violet in PBS. Colonies with at least 50 cells were automatically scored with the ImageJ software and the survival fraction was determined from the plating efficiency of the irradiated cells relative to the plating efficiency of the 0 Gy controls, as described in [4].

Western Blot (WB), Cellular fractionation and Co-Immunoprecipitation (CoIP). For WB on whole cell extracts, cells were lysed for 30' on ice in RIPA buffer (50 mM Tris-HCl pH 7.5, 150 mM NaCl, 0.5% Na deoxycholate, 1% NP40, 0.1% SDS) supplemented with 1 mM DTT, proteases and phosphatases inhibitors (Roche). Clarified lysates were then quantified by Bradford colorimetric assay (Biorad) and subjected to SDS-PAGE and western blot analysis following standard procedures.

For protein subcellular fractionation experiments, U2OS cells were irradiated with 10 Gy of IR or left untreated and then harvested at the indicated time points. Subcellular protein fractions were obtained with the Subcellular Protein Fractionation Kit (ThermoFisher #78840) following manufacturer's instructions. Nuclear soluble and chromatin-bound fractions were then loaded onto polyacrylamide gels and analysed by western blot.

For exogenous-endogenous CoIPs, HEK293T cells transfected with FLAG-tagged ZNF281, XRCC4 or empty vector or HEK293H cells stably expressing FLAG-DNA-PKcs were harvested and lysed for 30' on ice in CoIP buffer (10 mM Tris-HCl pH 8, 150 mM NaCl, 1 mM EDTA, 1% Triton X-100) supplemented with 1 mM DTT, proteases and phosphatases inhibitors (Roche). After centrifugation at maximum speed for 30' at 4°C, the supernatant was collected and incubated overnight with 25 μl of EZview™ Red ANTI-FLAG® M2 Affinity Gel (Sigma #F2426). The following day the resin was washed 5 times in CoIP buffer (for selected experiments salt concentrations of washing buffer were raised up to 250, 350, 450 or 600 mM NaCl) and then incubated for 30' at 4°C with 200 μg/ml of 3X FLAG peptide (Sigma #F4799) to release the immunoprecipitated proteins from the resin for competition. Recovered samples were then boiled with Laemmli buffer and loaded on polyacrylamide gels for western blot analysis.

For CoIPs among endogenous proteins, HEK293T or U2OS clarified lysates were incubated with 4 μg of the indicated antibodies overnight at 4°C rocking. On the following day, 25 μl of Pierce™ Protein A/G Magnetic

Beads (ThermoFisher #88802) were added to the mixture and incubated for 1h at 4°C. After extensive washes in CoIP buffer, proteins were released from beads through the addition of 2X Laemmli buffer and boiling. Protein lysates were then loaded on polyacrylamide gels for western blot analysis. All the antibodies used are listed in the Supplementary Table S2. Uncropped scans of all western blots are shown in Supplementary Fig. S6 and S7.

Immunofluorescence (IF). For γ-H2AX IF, cells were seeded on glass coverslips, treated as indicated and fixed with 4% paraformaldehyde for 10 min at room temperature (RT). For 53BP1 IF, cells were pre-extracted for 4 min with CSK buffer (100 mM NaCl, 300 mM sucrose, 3mM $MgCl_2$, 10 mM PIPES pH 6.8, 10mM β-glycerol phosphate, 50mM NaF, 1 mM EDTA, 1 mM EGTA, 5 mM Na orthovanadate, 0.5% Triton X-100) and then fixed with 4% paraformaldehyde on ice for 20 min. In both cases, after the fixation step, cells were permeabilized for 5 min in 0.25% Triton X-100 and blocked in 10% goat serum for 1h. Slides were then incubated with the indicated primary antibodies (Supplementary Table S2) 1h at RT and afterwards with secondary antibody (Alexa Fluor 488, invitrogen) along with Hoechst to counterstain nuclei. Images were acquired using a Zeiss LSM510 confocal microscope and analysis performed using the Volocity software (PerkinElmer). All images of a given experiment set were analysed under the same parameters.

Translocation assay. U2OS-AsiSI cells were induced with 4-OHT as indicated, and genomic DNA was extracted using the PureLink kit (Invitrogen). Illegitimate rejoining frequencies between DSBs induced in the same chromosome (MIS12::TRIM37) or in different chromosomes (TRIM37:: RBMXL1) were assessed by qPCR (Applied Biosystems® 7500 fast) or with endpoint PCR using Q5 High-Fidelity DNA Polymerase (New England Biolabs) according to the manufacturer’s instructions. Translocation frequencies were calculated using the $2^{-\Delta\Delta Ct}$ method, normalising on two control regions far from any AsiSI cut sites, as previously described [3]. Primers used for translocation assay are listed in the Supplementary Table S1.

Ligation-mediated cleavage assay. Quantification of the repair kinetics at AsiSI-induced breaks was performed as described [2] with minor modifications. Briefly, after in vivo induction of the AsiSI breaks, the genomic DNA was extracted with the PureLink™ Genomic DNA Mini Kit (ThermoFisher) and ligated with a biotinylated double-stranded oligonucleotide linker containing an AsiSI 3’ overhang. The pCMV6-Entry HIF1α plasmid linearized in vitro with AsiSI was spiked in each ligation mix and was used as internal control. After the ligation step, the DNA was fragmented for 2h with EcoRI, diluted in binding buffer (20 mM Tris-HCl pH 8.1, 0.1%

SDS, 1% Triton X-100, 2mM EDTA, 150 mM NaCl) and then incubated overnight at 4 °C in rotation with streptavidin magnetic beads (Dynabeads™ MyOne™ Streptavidin C1, ThermoFisher). Beads were then washed five times in washing buffer (50 mM Tris-HCl pH 8.1, 0.1% SDS, 500 mM NaCl) and twice in TE (10 mM Tris-HCl pH 7.5, 1mM EDTA) before digestion with HindIII for 4h at 37 °C. Pulled-down DNAs and their respective inputs were precipitated with glycogen, sodium acetate pH 5.2 and ethanol and then resuspended in nuclease-free water and analysed by qPCR (Applied Biosystems® 7500 fast). Primers used for this assay are listed in the Supplementary Table S1.

NHEJ and HR repair reporter assays. Fluorescence-based NHEJ and HR reporter assays [6] were used to quantify the efficiency of DSBs repair. U2OS cells harbouring NHEJ or HR reporter cassettes were reverse silenced with the indicated siRNAs. Nucleofection was performed 48h later with 2.3μg I-SceI plus 200ng mCherry encoding plasmids using Amaxa SE Cell Line 4D Nucleofector Kit (Lonza). Cells were collected after 48-72h and analysed by flow cytometry. The DSBs repair efficiency was calculated as the ratio between the percentage of GFP (successfully repaired DSBs) and mCherry (transfection efficiency) positive cells.

Laser micro-irradiation and live cell imaging. U2OS cells expressing the indicated fluorescent-fusion proteins were seeded into Lab-Tek chambered coverglass wells (#155383, Thermo Scientific) and pre-sensitized with 20 μM Hoechst 33342 (Thermo Scientific) for 10 min at 37 °C prior the UVA laser micro-irradiation. DNA damage was induced focally using the 3i 'Ablate' UV 355 nm pulsed laser system in a circle of radius 1 μm. Image acquisition was performed using a spinning disk inverted microscope system (3i), while cells were maintained at 37 °C and 5% CO_2 in a stage top environmental control system (okolab) for the whole duration of the experiment. The 488 nm (for EGFP-constructs) or the 561 nm (for mCherry-constructs) channels were imaged every 5 s to monitor the fluorescence intensity. Background subtraction and photobleaching correction were applied using the 3i Slidebook software and, for each cell, relative fluorescence intensity was calculated by normalising the intensity of pre-damaged cells (t=0s) to a value of 1.

Site-directed DNA damage induction and Chromatin immunoprecipitation (ChIP). U2OS cells were infected with a lentivirus expressing the dd-HA-ER-I-PpoI [5] endonuclease for 24h in presence of 8 μg/ml of polybrene. To enrich the I-PpoI-containing population, cells were subjected to a 24h round of puromycin selection (2.5 μg/ml). Once the puromycin was washed out, cells were allowed to recover for additional 24h. To

activate the endonuclease and to prevent its degradation, cells were treated with 1 μM Shield1 (Takara) for 3h and then with 1 μM 4-hydroxytamoxifen (4-OHT) (Sigma) for 2h. Cells were then crosslinked for 10 min at RT with 1% paraformaldehyde and ChIP assay was performed using the MAGnify Chromatin Immunoprecipitation System (Invitrogen), following the manufacturer's instructions. PCR was carried out using REDTaq® ReadyMix™ PCR Reaction Mix (Sigma) according to the manufacturer's instructions by using the primers listed in the Supplementary Table S1.

GDC (Genomic Data Commons) TCGA (The Cancer Genome Atlas) datasets analysis. Kaplan-Meier survival curves were generated using raw data of the sarcoma (GDC TCGA SARC), colon adenocarcinoma (GDC TCGA COAD), melanoma (GDC TCGA SKCM) and lung adenocarcinoma (GDC TCGA LUAD) datasets downloaded from the UCSC Xena Browser (https://xenabrowser.net/heatmap/; https://www.biorxiv.org/content/early/2018/05/18/326470).

Statistical analysis. Statistical analysis was performed using the Student's *t*-test (for clonogenic, reporter, translocation and ligation assays), the Mann–Whitney test (for non-parametric testing of the number of γ-H2AX foci per cell) or the Log-rank Mantel-Cox test (for Kaplan-Meier survival curves) using Graphpad Prism (version 6, Graphpad Software Inc., USA). The test used is indicated in figure legends and in all cases, *p ≤ 0.05, **p ≤ 0.01, ***p ≤ 0.001.

SUPPLEMENTARY REFERENCES

1 Aymard F, Bugler B, Schmidt CK, Guillou E, Caron P, Briois S *et al*. Transcriptionally active chromatin recruits homologous recombination at DNA double-strand breaks. Nat Struct Mol Biol 2014; 21: 366-374.

2 Chailleux C, Aymard F, Caron P, Daburon V, Courilleau C, Canitrot Y *et al*. Quantifying DNA double-strand breaks induced by site-specific endonucleases in living cells by ligation-mediated purification. Nat Protoc 2014; 9: 517-528.

3 Cohen S, Puget N, Lin YL, Clouaire T, Aguirrebengoa M, Rocher V *et al*. Senataxin resolves RNA:DNA hybrids forming at DNA double-strand breaks to prevent translocations. Nat Commun 2018; 9: 533.

4 Franken NAP, Rodermond HM, Stap J, Haveman J, van Bree C. Clonogenic assay of cells in vitro. Nat Protoc 2006; 1: 2315-2319.

5 Goldstein M, Derheimer FA, Tait-Mulder J, Kastan MB. Nucleolin mediates nucleosome disruption critical for DNA double-strand break repair. Proc Natl Acad Sci U S A 2013; 110: 16874-16879.

6 Seluanov A, Mao Z, Gorbunova V. Analysis of DNA double-strand break (DSB) repair in mammalian cells. J Vis Exp 2010.
